# Ethosuximide inhibits acute histamine- and chloroquine-induced scratching behavior in mice

**DOI:** 10.1186/s13041-021-00762-1

**Published:** 2021-03-02

**Authors:** Vinicius M. Gadotti, Gerald W. Zamponi

**Affiliations:** Department of Physiology and Pharmacology, Hotchkiss Brain Institute, Alberta Children’s Hospital Research Institute, Cumming School of Medicine, University of Calgary, Calgary, AB Canada

**Keywords:** Ethosuximide, Cav3.2 channel, Itch, Histamine, Chloroquine

## Abstract

We have recently reported that the Cav3.2 T-type calcium channel which is well known for its key role in pain signalling, also mediates a critical function in the transmission of itch/pruritus. Here, we evaluated the effect of the clinically used anti-seizure medication ethosuximide, a well known inhibitor of T-type calcium channels, on male and female mice subjected to histaminergic- and non-histaminergic itch. When delivered intraperitoneally ethosuximide significantly reduced scratching behavior of mice of both sexes in response to subcutaneous injection of either histamine or chloroquine. When co-delivered subcutaneously together with either pruritogenic agent ethosuximide was also effective in inhibiting scratching responses in both male and female animals. Overall, our results are consistent with an important role of Cav3.2 T-type calcium channels in modulating histamine-dependent and histamine-independent itch transmission in the primary sensory pathway. Our findings also suggest that ethosuximide could be explored further as a possible therapeutic for the treatment of itch.

## Introduction

Pruritus significantly influences quality of life measures, such as mood, the ability to concentrate, and sleep, and impacts quality of life more than many other skin conditions [1, 2]. Similar to painful stimuli, pruritogens activate peripheral sensory neurons that then communicate in the spinal cord with second order neurons that project to brain regions where itch is perceived as an unpleasant sensation [3]. Distinct but perhaps partially overlapping populations of neurons are involved in transmitting pain and itch related information, however, there is accumulating evidence that both pain and itch transmitting neurons express T-type calcium channels [4–7]. Based on their biophysical properties, T-type calcium channels are uniquely suited towards regulating neuronal excitability. The mammalian genome encodes three distinct T-type calcium channel isoforms, Cav3.1, Cav3.2 and Cav3.3, with Cav3.2 having been prominently associated with peripheral pain transmission [5]. We have recently explored the putative role of Cav3.2 channels in the transmission of histamine and chloroquine-dependent itch [7]. We found that Cav3.2 null mice were almost completely resistant to the actions of both histamine and chloroquine. Furthermore, subcutaneous injection of an experimental small molecule inhibitor of Cav3.2 channels significantly attenuated scratching responses to injection of histamine or chloroquine in wild type mice. These data collectively suggested that Cav3.2 channels mediate an important role in itch responses in the skin, presumably by controlling action potential initiation in peripheral nerve endings.

Ethosuximide (Zarontin) is an archetypal T-type calcium channel therapeutic that is used clinically to treat absence epilepsy, particularly in children [8]. Given that ethosuximide is generally well tolerated and thought to have few drug interactions [9, 10], we surmised that this molecule could perhaps be used to treat itch. Therefore, as a first step, we wanted to obtain preclinical evidence of possible anti pruritonergic effects of locally and systemically delivered ethosuximide in mouse models of acute itch. Here, we report that ethosuximide potently inhibits scratching responses associated with subcutaneous delivery of histamine or chloroquine in both male and female mice.

## Methods

Experiments were carried out on adult (7–11 weeks old) male or female C57BL/6J mice purchased from Jackson laboratories following approval by the institutional Animal Care Committee. Mice were housed at five per cage (30 × 20 × 15 cm) with free access to food and water. The housing room was kept at a temperature of 23 ± 1 °C and under a 12-h light/dark cycle (lights on at 7 am). All experiments were carried out between 9 am and 3 pm. The pruritogens chloroquine diphosphate (Sigma-Aldrich) and histamine (Sigma-Aldrich), as well as ethosuximide (Sigma-Aldrich), were dissolved in PBS solution. When compounds were delivered subcutaneously (s.c.), a standard low volume of 20 μl was injected.

The protocols for induction of either histaminergic or non-histaminergic itch were performed according to our previous work [7]. Briefly, volumes of 20 µl of histamine or chloroquine were given subcutaneously (s.c.) at the back of the neck using a BD micro-fine 28 g 1/2 insulin syringe. Animals had the fur on their back trimmed 48 h before the experiments. Mice were placed individually into plexiglass observation chambers and the total time spent scratching the injected site was scored for 30 min beginning immediately after the administration of either pruritogen. Mice were treated systemically with ethosuximide (10–30 mg/kg, i.p.) 0.5 h before receiving histamine or chloroquine. When ethosuximide was co-delivered with a pruritogen, volumes of 20 µl of a solution containing an association of ethosuximide (100–1000 μg) and histamine (100 μg) were injected.

Data were analyzed with Graphpad Prism 6.0 and Graphpad InStat 3.0 and are presented as the mean ± SEM. One-way analysis of variance (ANOVA) with Tukey’s post hoc correction was used. Statistical significance was accepted at the level of p < 0.05.

## Results and discussion

**Fig. 1:**
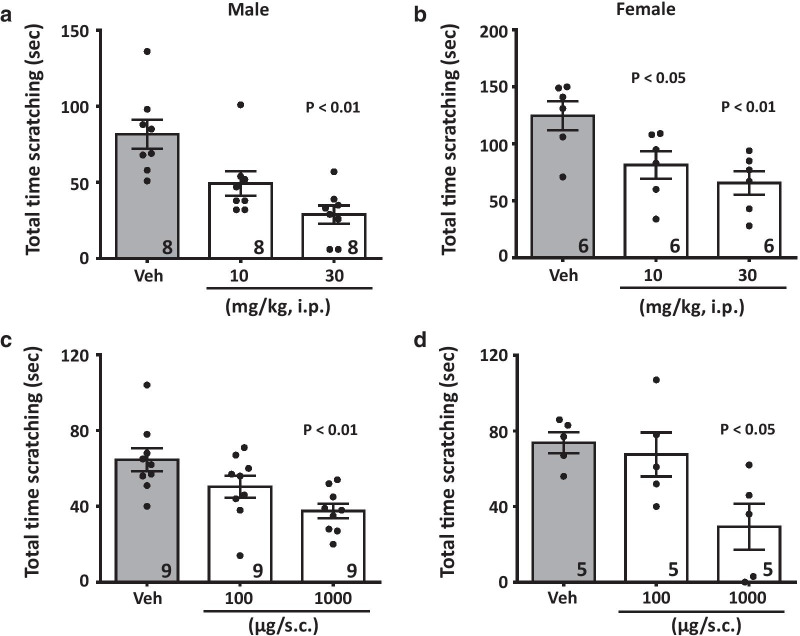
Ethosuximide inhibits acute itch responses induced by histamine. Dose-dependent inhibition of the total time scratching the nape in (**a**, **c**) male or (**b**, **d**) female mice by ethosuximide when delivered (**a**, **b**) systemically (10.0–30.0 mg/kg, i.p.) before histamine treatment, or (**c**, **d**) when co-delivered (10.0–100.0 μg/co-injected) with histamine. Each bar represents the mean ± S.E.M. and is representative of two or three experimental runs. Numbers shown in the bars reflect numbers of mice. One-way ANOVA reveals significant differences

**Fig. 2:**
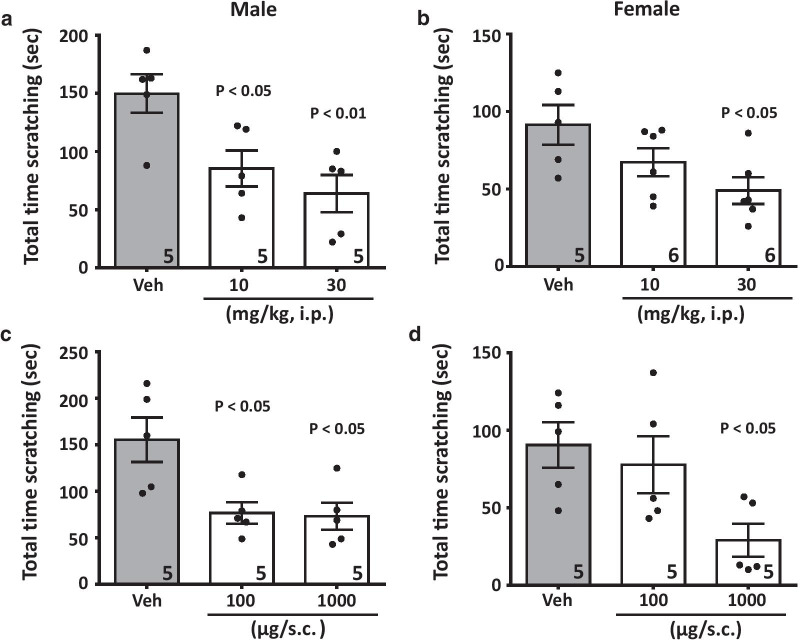
Ethosuximide decreases scratching behavior induced by chloroquine. Dose-dependent inhibition of the total time scratching the nape in (**a**, **c**) male or (**b**, **d**) female mice by ethosuximide when delivered (**a**, **b**) systemically (10.0–30.0 mg/kg, i.p.) before chloroquine treatment, or (**c**, **d**) when co-delivered (10.0–100.0 μg/co-injected) with chloroquine. Each bar represents the mean ± S.E.M. and is representative of two experimental runs. Numbers shown in the bars reflect numbers of mice. Two-way ANOVA reveals statistical differences

We tested whether ethosuximide is effective against histaminergic and non-histaminergic itch by its ability to inhibit scratching behavior caused by either histamine (100 μg) or chloroquine (200 μg) injected subcutaneously (20 μl) into the nape of the animals. Intraperitoneal pre-treatment (30 min prior) of mice with ethosuximide (10.0–30.0 mg/kg) produced a dose-dependent inhibition of itch responses in male (Fig. 1a) and female (Fig. 1b) mice injected with histamine. Ethosuximide was also able to decrease the time mice spent scratching when co-delivered subcutaneously (100.0–1000.0 μg) with histamine in either male (Fig. 1c) or female (Fig. 1d) mice. Similarly, systemically delivered ethosuximide (10.0–30.0 mg/kg) elicited dose-dependent inhibition of scratching behaviour of male (Fig. 2a) and female (Fig. 2b) subjects that received chloroquine diphosphate. Likewise, ethosuximide produced a significant reduction of itch responses when co-delivered (100.0–1000.0 μg) with chloroquine in male (Fig. 2c) and female (Fig. 2d). Mice receiving pruritogens did not display flinching or vocalization which are behaviors that would typically indicate nociceptive or nocifensive responses rather than itch [11]. Together, our data indicate that a clinically used T-type calcium channel inhibitor attenuates scratching after either local or systemic delivery.

More than one in ten people suffers from chronic itch [12] and the global Pruritus Therapeutics market was valued at USD 11 Billion in 2018. In many cases, chronic itch is poorly managed and leads to poor quality of life, impaired sleep and comorbidities such as depression [1, 2, 13, 14]. This is particularly the case for patients on dialysis who frequently suffer from uremic itch, with about 40% of patients with end-stage renal disease being afflicted with moderate to severe pruritus [15]. In this context it is interesting to note that Cav3.2 channel expression is upregulated in the skin of uremic itch patients (presumably in nerve endings) [16], hinting at a possible role of these channels in uremic itch pathology. While we did not examine mouse models of chronic pruritus here, there is accumulating evidence that Cav3.2 channels are involved in the development of histamine and non-histamine dependent itch in rodents. It has been reported that Cav3.2 channels are important for hydrogen sulfide induced itch responses [6] in mice, and our recently published work has shown that Cav3.2 channels are critical for histamine and chloroquine induced itch in male and female mice [7]. Although the cell signaling pathways for histamine and non-histamine dependent itch may be distinct [17], they appear to both involve Cav3.2 channels which is reminiscent of their role in the transmission of peripheral pain signals. Our observation that ethosuximide is effective when applied locally suggests that Cav3.2 channels are important for the activity of sensory nerve endings in the skin. In this context, it is possible that pruritogens may enhance Cav3.2 channel activity per se, or alternatively the role of Cav3.2 channels may be to maintain neuronal activity that is triggered by pruritogen actions on other channels and receptors. In either case, inhibition of Cav3.2 channels would be expected to suppress itch signaling.

Systemically delivered ethosuximide readily crosses the blood brain barrier, and it is thus possible that Cav3.2 and perhaps other Cav3 channel isoforms may be involved in the transmission/processing of itch signals at the level of the spinal cord and/or brain. Dorsal root ganglia which are also accessible to intraperitoneally administered ethosuximide are known to express high levels of Cav3.2 calcium channels [18, 19], but may also express other Cav3 family members. It is known that that ethosuximide inhibits all three T-type calcium channel isoforms [20]. Hence, despite our previous findings showing that Cav3.2 null mice are resistant to itch [7], we cannot rule out the possibility that the effect of ethosuximide on scratching responses might also involve inhibition of Cav3.1 or Cav3.3 channels. From a mechanistic point of view, it will be interesting to determine whether spinally delivered ethosuximide has antipruritonergic effects, although from a clinical vantage point, the fact that systemic delivery was effective is much more important.

We reiterate that we did not examine the effects of ethosuximide in models of chronic pruritus. Nonetheless, our data may set the stage for further preclinical studies in mouse models of chronic itch, and perhaps clinical studies that examine the possible utility of ethosuximide as a therapeutic for chronic itch sufferers. We note that ethosuximide was recently evaluated in a randomized clinical trial for neuropathic pain, and not only failed to show efficacy, but at the doses used, resulted in a number of adverse effects that required the termination of the trial [21]. This stands at odds with the notion that ethosuximide is generally well tolerated by epilepsy patients [10]. It remains to be determined whether ethosuximide could have a wider therapeutic window for itch compared to neuropathic pain, or alternatively, one may be able to explore transdermal effects of topically formulated ethosuximide as a possible anti itch agent.

Altogether, our data further support an important function of T-type calcium channels in the development of different forms of itch, and may offer possible new therapeutic pathways.

## Data Availability

All data generated or analysed during this study are included in this published article.
